# Reflections on the Health System Impact Fellowship and the Future of Embedded Research: Comment on "Early Career Outcomes of Embedded Research Fellows: An Analysis of the Health System Impact Fellowship Program"

**DOI:** 10.34172/ijhpm.8615

**Published:** 2024-10-28

**Authors:** Elena Lopatina, Deepa Singal, Kiran Pohar Manhas

**Affiliations:** ^1^Alberta Health Services and Department of Community Health Sciences, Cumming School of Medicine, University of Calgary, Calgary, AB, Canada.; ^2^Autism Alliance of Canada and Department of Pediatrics, Faculty of Medicine & Dentistry, College of Health Sciences, University of Alberta, Edmonton, AB, Canada.

**Keywords:** Embedded Research, Embedded Research Training, Health Services Research, Learning Health Systems

## Abstract

The Health System Impact (HSI) Fellowship program in Canada offers a transformative approach to health services and policy research (HSPR) training, preparing PhD graduates for diverse career pathways and leadership roles within learning health systems. This commentary builds on Kasaai and colleagues’ evaluation of the HSI Fellowship to discuss the diverse career paths of alumni and highlight the multifaceted benefits of the program. Further, we emphasize the need for future research and knowledge mobilization to better understand and evaluate embedded research roles. Developing a robust evaluation framework is essential to capture the unique impacts of embedded research, fostering a culture that prioritizes and integrates it, thereby driving the transformation towards learning health systems.

## Introduction

 As detailed in “Early Career Outcomes of Embedded Research Fellows: An Analysis of the Health System Impact Fellowship Program” by Kasaai et al,^1^ Canada’s Health System Impact (HSI) Fellowship program provides a transformative approach to health services and policy research (HSPR) training. This program aims to prepare PhD graduates for diverse career pathways and leadership roles within learning health systems. Kasaai et al present the benefits of the HSI Fellowship, emphasizing its positive impact on career readiness and the employability of alumni.^1^

 This commentary builds on peer-reviewed literature and our personal experiences as HSI Fellowship alumni and embedded scientists to discuss Kasaai and colleagues’ findings on the diverse career paths of HSI Fellowship alumni within the rapidly evolving field of embedded research. We emphasize the multifaceted benefits of the HSI Fellowship program, highlighting its advantages for host organizations and its contributions to advancing embedded research. Furthermore, we underscore the need for future research and knowledge mobilization to advance the employ and impact of embedded researchers, which is crucial to evolve learning health systems.

## Diverse Career Paths of the HSI Fellowship Alumni

 Kasaai et al highlight the diverse career paths of HSI alumni, who pursue careers across various sectors, including academia, health system organizations, government agencies, and non-profit organizations.^1^ This diversity reflects the broad field of embedded research and the varied needs of organizations.^2^ Post-fellowship employment outcomes generally aligned with the initial career aspirations expressed at the start of the HSI Fellowship.^1^ This alignment and the diversity of career paths underscore the value of a novel HSPR training approach that enables fellows to straddle and learn from academic and host-partner organizations. The flexibility of organizations that fall within the HSI purview and the diversity of HSI projects fellows pursue generate flexibility that caters to the unique aspirations and career goals of its myriad participants.

 The authors also discuss potential reasons for instances where post-fellowship employment outcomes do not align with initial career aspirations.^1^ These reasons include the evolution of individuals’ career preferences, expanding networks that offer new opportunities, and the short length of time between fellowship completion and the study’s career tracking.^1^ Other reasons for misalignment, not discussed in detail by Kasaai et al, could include the novelty and variability of embedded researcher roles. Also, not all host-partner organizations are large institutions or systems; burgeoning host-partner organizations are not-for-profits that may lack the budgetary capacity to create full-time, long-term positions that many alumni prioritize in their post-fellowship careers.

 Post-fellowship, many HSI alumni secure positions that did not previously exist at their host organizations. The flexibility to influence future positions and the experience of working with both a host-partner organization and an academic institution during the HSI Fellowship may lead aspiring embedded scientists to take on hybrid roles, recognizing the potential benefits of such positions. Moreover, since embedded researcher roles can be implemented in various settings and can bridge or blur the boundaries between academia and policy/practice in multiple ways,^2^ hybrid roles could also be considered a variation of these positions.

 Much of the literature to date has defined embedded researchers in a rather narrow manner.^2^ However, a better understanding of the wide range of roles, activities, identities, and affiliations related to embedded researchers is emerging.^2^ In the future, the growing body of knowledge and the increased awareness of embedded researcher roles both among HSPR graduates and health system organizations could lead to a better alignment of employment outcomes with initial career aspirations.

## Multidirectional Benefits of the HSI Fellowship

 As suggested by Kasaai et al and previously discussed by other authors, the benefits of the HSI Fellowship are multifaceted and multidirectional, positively impacting the fellows, the host health system organizations, and the field of embedded research overall.^1,3-7^ For the fellows, working within health system organizations provides invaluable hands-on experience, exposure to real-world challenges and solutions, and the opportunity to develop professional skills and expertise often not covered in graduate studies.^3,4,6,7^ This experience enhances their ability to apply theoretical knowledge to practical problems, preparing them for impactful career.^1,3,4^

 The HSI Fellowship benefits the health system organizations by building their research capacity and understanding of what is involved in supporting, and benefiting from, graduate-trained HSPR fellows.^5,8-10^ Fellows provide research and analytic skills, and also through engagement and collaboration, they build such skills in organizational staff and partners. Kasaai et al indicate that 22% of HSI Fellowship alumni were hired by the same health system organization where they completed their fellowship.^1^ This statistic underscores the value that host organizations place on the fellows’ contributions and the benefits derived from the embedded research conducted during the fellowship.

 Hosting an HSI fellow allows organizations to experience the benefits of embedded research firsthand, which can encourage them to prioritize it in their future work. As a result, more organizations may consider creating positions for HSI alumni and other HSPR graduates interested in working in embedded settings. The fact that many HSI alumni are hired by their host organizations^1^ and that many organizations have served as hosts multiple times demonstrates the ongoing interest in embedded research among host organizations. The HSI Fellowship not only prepares HSPR graduates for impactful careers and helps host organizations address their pressing challenges, but also contributes to the evolution of vacancies in the field, advancing the field of embedded research overall.

## Future Research and Knowledge Dissemination to Advance Embedded Research and Learning Health Systems

 Nowadays in the Canadian context, the ultimate goal of embedded HSPR research and training programs like the HSI Fellowship is to move towards learning health systems in practice.^11^ Learning health systems are characterized by their ability to continuously generate and apply evidence to improve outcomes.^11,12^ Embedded research plays a crucial role in this transformation by bridging and blurring the gap between research and operations, thus, facilitating the rapid translation of evidence into action.^4,11^ However, given the existing knowledge gaps, future research is needed to better understand embedded research roles and develop approaches to evaluate the contributions of embedded researchers.^2,4,5^

 Smith and Johnson described the barriers and facilitators to implementing embedded research in health settings in a systematic review (n = 13 articles).^13^ Further research on the HSI Fellowship, and other embedded research endeavours, should quantify the impact of these barriers and facilitators, which included leadership, organizational culture, capabilities, education, knowledge, management, time, culture, and resources.^13^ In particular, understanding the presence of which facilitators or absence of barriers in the 22% of organizations wherein fellows remained employed post-fellowship could inform capacity building across diverse health system organizations.

 Future studies building on Kasaai et al should explore the long-term career outcomes of HSI Fellowship alumni to further understand the skills and expertise required to advance learning health systems and define the roles of embedded researchers. Reaching out to alumni themselves (vs. using social media) would add valuable insights on career trajectories and decision-making and would limit potential bias due to social media profiles not being up-to-date. Research on the outcomes of HSI Fellowship projects and host organizations’ experiences can provide valuable insights into evaluating embedded roles and measuring their impact and effectiveness.

 There is also a need for increased knowledge mobilization of research on the HSI Fellowship, and other embedded research endeavours, directly to host-partner organizations and academic institutions to promote a culture that values and supports embedded research. Disseminating these findings in academia offering graduate HSPR programs will raise awareness of the diverse career possibilities available to graduates. It could spur academia to modernize its graduate-level training for even better preparedness for novel or hybrid roles. It can educate about the roles of embedded researchers, thereby challenging the existing hierarchy in research, where often only academic researchers are viewed as having the skills and ability to generate robust evidence and drive a collaborative research agenda.^2^

 Embedded researchers have a unique opportunity for higher impact because they work within the system, bridging the gap between research and practice. However, establishing and measuring the outcomes of embedded research can be challenging.^2,5,8^ While frameworks exist for evaluating the impact of academic research, similar frameworks for embedded research are lacking.^2^ As a result, health system organizations, universities, research institutes, and funding agencies may struggle to appropriately recognize and reward the contributions of embedded researchers.^5^

 Traditional academic metrics like publications and grants do not adequately reflect the value and impact trajectories of embedded research.^5^ Therefore, the next logical step is to develop a dedicated evaluation framework tailored to the distinct nature of embedded research. This framework should identify key indicators of research success in embedded settings, such as influencing policy and practice changes that lead to improved health outcomes and enhanced performance of health system organizations, building organizational capacity for rapid learning and improvement, and effectively translating evidence into practice.^5,11^ Aligning the evaluation metrics with the enriched core competency framework for HSPR^7^ can help delineate the unique contributions of embedded researchers, making their impact more visible and understandable to different audiences.

 Developing this dedicated framework would not only demonstrate the impact of embedded research but also ensure that its value is recognized and rewarded appropriately. Furthermore, such a framework could inform capacity-building within organizations, guiding them on how to effectively integrate embedded research into their operations. By clearly articulating the benefits and impacts of embedded research, the framework would encourage more organizations to hire embedded scientists and create additional positions for HSI alumni and other HSPR graduates, advancing the field of embedded research and learning health systems.

## Conclusion

 The HSI Fellowship exemplifies how strategic training programs can prepare a strong cadre of embedded researchers. Future research and knowledge dissemination are essential to fully realize the potential of embedded research and training programs such as the HSI Fellowship. It is crucial to develop a robust evaluation framework to capture the unique impacts of embedded research. Such a framework will not only demonstrate the value of embedded research but also foster a culture that prioritizes and integrates it, ultimately driving the transformation towards learning health systems.

## Ethical issues

 Not applicable.

## Conflicts of interest

 Authors declare that they have no conflicts of interest.

## Disclaimer

 EL and DS hold the Health System Impact Embedded Early Career Researcher Awards from the Canadian Institutes of Health Research. EL is an alumna of the Health System Impact Doctoral and Postdoctoral Fellowships. DS and KPM are alumni of the Health System Impact Postdoctoral Fellowship.
